# Language switching may facilitate the processing of negative responses

**DOI:** 10.3389/fpsyg.2022.906154

**Published:** 2022-09-06

**Authors:** Anqi Zang, Manuel de Vega, Yang Fu, Huili Wang, David Beltrán

**Affiliations:** ^1^Instituto Universitario de Neurociencia, Universidad de La Laguna, Santa Cruz de Tenerife, Spain; ^2^School of Foreign Languages, Zhejiang University City College, Hangzhou, China; ^3^Departamento de Psicología Básica I, Universidad Nacional de Educación a Distancia (UNED), Madrid, Spain

**Keywords:** language switching, negation processing, inhibitory mechanism, cognitive control, bilinguals

## Abstract

It has been proposed that processing sentential negation recruits the neural network of inhibitory control (de Vega et al., 2016; Beltrán et al., 2021). In addition, inhibition mechanisms also play a role in switching languages for bilinguals (Kroll et al., 2015). Since both processes may share inhibitory resources, the current study explored for the first time whether and how language-switching influences the processing of negation. To this end, two groups of Spanish-English bilinguals participated in an encoding-verification memory task. They read short stories involving the same two protagonists (Montse and Jordi), referring to their activities in four different scenarios in Spanish or English. Following each story, the participants received verification questions requiring “yes” or “no” responses depending on whether a given fact was correctly referred to one of the protagonists. Some of the verification questions were in the story’s original language (non-switch condition) and others in the alternate language (switch condition). Results revealed that language-switching facilitated negative responses compared to affirmative responses, exclusively for questions switching from dominant language (L1) to non-dominant language (L2). This effect might reflect that the domain-general mechanisms of inhibitory control are recruited at least partially for both language switch and negation process simultaneously, although this phenomenon is modulated by language dominance.

## Introduction

Imagine you are a Spanish-English bilingual, you read a news item from a Spanish newspaper and then you share the news item with two friends, one a Spanish monolingual and the other an English monolingual. After telling them the news, they began to ask you many details in Spanish or English, such as “Who had the accident? Mary?” and you replied “No,” or “>Quién conducía el coche? >Peter?” [Who was driving the car? Peter?] and you answer “Sí” [Yes]. This is an example of how learning and retrieving linguistic information can rely on the same language (L1 – L1), or on a different language (L1 – L2) to produce an affirmative or negative answer in the appropriate language. In this situation, you retrieve information in the original language, but, to answer some questions, you eventually need to switch to another language. To do that, one remarkable ability of bilinguals is cognitive control or monitoring to discern between their two languages and select one of them while suppressing the other to minimize the interference. Numerous studies have investigated how language control plays a crucial role in bilinguals (Green, 1998; Meuter and Allport, 1999; Abutalebi and Green, 2008), giving prominence to the demands for inhibitory resources. Yet, cognitive control and inhibition are also important for many aspects of language processing, including comprehension and production of negation (de Vega et al., 2016; Beltrán et al., 2019; Liu B. et al., 2020; Dudschig et al., 2021; Vitale et al., 2021). Therefore, the bilingual scenario above raises some questions: Do language switching and negative responses share the same inhibitory resources? And if so, how does the former influence the latter?

The role of cognitive control in bilinguals has been studied with the so-called language-switching paradigm. This paradigm requires the naming of pictures or digits in either of two languages, depending on explicit cues (Meuter and Allport, 1999), a pre-ordered sequence (Declerck et al., 2013), or a voluntary selection (Liu H. et al., 2020). The typical finding is that switching from one language to another requires a longer response time than keeping the same language (Costa and Santesteban, 2004; Declerck et al., 2013; Declerck and Philipp, 2015b). Many researchers have reported an asymmetric switching cost effect, with larger switching costs to shift from less dominant (L2) to dominant language (L1) than to shift from dominant (L1) to less dominant language (L2), which has been attributed to differential demands on inhibitory control (De Bot, 1992; Green, 1998; Meuter and Allport, 1999). That is, producing in a specific language involves inhibition of the non-target language, but more inhibition is needed to suppress the irrelevant L1 when producing L2 (see a review, Gade et al., 2021).

Most of the research in this field has focused on bilinguals’ production of isolated words. However, some research also reported language switching effects on comprehension (Bultena et al., 2015; Wang, 2015) and long-term memory (Marian and Neisser, 2000; Matsumoto and Stanny, 2006; Marian and Kaushanskaya, 2007). In general, according to the encoding specificity principle, matching features of encoding and retrieval contexts facilitates recall, in comparison with mismatching encoding and retrieval contexts (see Tulving and Thomson, 1973; Davies and Thomson, 1988; for a review). Applying this principle to the field of bilingualism, several studies have examined language-dependent effects on memory, typically reporting that recall is better when the language of retrieval matched the language of encoding rather than when they are mismatched. This language matching advantage occurs in autobiographical memory (Marian and Neisser, 2000; Matsumoto and Stanny, 2006), semantic memory for world knowledge (Marian and Kaushanskaya, 2007), academic-type memory (Marian and Fausey, 2006), and narrative stories (Wang, 2022).

Negation processing has often been associated with the suppression of negated information (see Beltrán et al., 2021 for a recent overview). Indeed, several lines of research support the hypothesis that negation has inhibition-like effects. One approach reveals that negation modulates embodied effects during the comprehension of action language, as in the case of the reduction of motor interference effects in behavioral studies (Aravena et al., 2012; Bartoli et al., 2013; García-Marco et al., 2019), and the reduction of activation of the motor and premotor cortex reported by neuroimaging studies with fMRI technique (Tettamanti et al., 2008; Tomasino et al., 2010). Similarly, non-invasive brain stimulation studies identified a larger cortical silence period (a measure of inhibition in the GABAergic system) associated with negation when single-pulse TMS (Transcranial Magnetic Stimulation), which was used for stimulating peripheral nerves with a similar mechanism of activation as for electrical stimulation (Terao and Ugawa, 2002), was applied to M1 during the comprehension of action verbs (Papeo et al., 2016). Another approach uses the probe recognition paradigm to assess the activation level and the recall performance for negated concepts compared to affirmed concepts. The typical results showed longer latencies and higher error rate for negated concepts compared to non-negated concepts, indicating less accessibility for negated concepts, probably because negation interferes with (or inhibits) conventional concept encoding in working memory (e.g., MacDonald and Just, 1989; Kaup and Zwaan, 2003; Mayo et al., 2004; Orenes et al., 2014). Finally, EEG studies have demonstrated that negation recruits mechanisms of inhibitory control (de Vega et al., 2016; Beltrán et al., 2018, 2019; Liu B. et al., 2020). For instance, de Vega et al. (2016) provided the first evidence that understanding negative action sentences interacts with the processes required to suppress a prominent motor response in a concurrent Go/NoGo task, modulating the frontal theta rhythm, which is considered a typical marker of response inhibition.

The above studies mainly focus on the processing of sentential negation, that is, how sentences with a negative marker are understood. Yet, people produce negations as much as understand them. Thus, developmental studies have shown that children begin to use negative responses (no/not) very early, during the second year of life, to reject an object, or to stop or prevent an imminent action, establishing thus a strong association between the verbal markers of negation and the rejection and prevention of an action. Moreover, in this early stage of linguistic development, the child often use negation for self-prohibition, when she is about to engage in a forbidden action (e.g., Bloom, 1970; Pea, 1980; Choi, 1988). In fact, we can assume that inhibitory control underlies production of negations since the early childhood.

One important pragmatic function of negations is denial (Bloom, 1970). Denial occurs when a negative utterance is produced in response to a question that refers to a false content; for example, responding “no” when asked “Is this work written in Spanish?” Interestingly, verification tasks involving affirmative or negative responses (denials) have been widely used in studies of language and memory. Typically, participants receive statements referred to semantic memory contents (“Do cats eat vegetables?”), world knowledge (“Has Donald Trump been president?”), pictures or episodic memories about previously learned content and they simply have to answer yes or no. In a pioneering study, Craik and Tulving (1975) utilized a memory retrieval paradigm to identify distinct effects of response polarity on memory in their study depending on levels of processing. Participants had to initially encode words at various levels of processing, such as whether they were written in capital letters (shallow encoding) or whether they fit into a semantic category or sentence structure (deep encoding). In a posterior incidental memory test, they found that negative (no) responses had poorer recall than affirmative (yes) ones, particularly under deep encoding circumstances, supporting the hypothesis that negation might induce forgetting by weakening encoding strength. A few behavioral investigations have shown that the impact of negation on the encoding process persists over time, impairing long-term recall of negated information (Cornish and Wason, 1970; Craik and Tulving, 1975; Fiedler et al., 1996; Mayo et al., 2014; Zhang et al., submitted)^1^. For example, Mayo et al. (2014) reported the first comprehensive demonstration of the negation-induced amnesia effect. They found that actively negating a feature of an entity induced more memory loss of the entity itself compared to affirming the feature by conducting four tests in which they showed participants either short videos (Experiments 1–3) or verbal narratives (Experiment 4) embedded in a four-phase memory paradigm: study phase, verification task, distractive, and an incidental later free recall task. This negation-induced amnesia effect could be attributed to the short-term inhibitory effect of negation during the first memory test. Therefore, negation manipulates the encoding process to induce later forgetting in the retrieval phase.

Most research on the inhibitory effects of negation has been conducted with monolingual participants, while the processing of negation by bilinguals received little attention. Previous research on negation in bilinguals is generally driven by the idea that negation is universal and the processing of negation is more complicated than processing affirmation, regardless of the language (e.g., Hasegawa et al., 2002). Yet, since bilinguals have constant exercise to regulate the two languages they use, showing a stronger ability to resolve response conflict in non-linguistic activities (Bialystok et al., 2004; Costa et al., 2008), the study of negation processing in bilinguals may shed lights on the underlying mechanism of negation. To this end, the current study aimed to investigate the inhibitory effect of negation in a memory retrieval paradigm for bilinguals.

This study aims to explore the impact of language-switching on the processing of negation in an encoding-retrieval memory task. To this end, an online behavioral experiment was conducted with two groups of unbalanced Spanish-English bilinguals. One group of participants initially read stories in Spanish (L1), and the other group read the same stories in English (L2). Immediately after reading each story, the participants received a set of verification questions about the story contents, requiring a “yes” or “no” response. Some of the questions for verification were presented in the original language of the story (non-switch condition) and others in the alternative language (switch condition). In other words, the two critical manipulations of response polarity (affirmative vs. negative) and language sequence (switch vs. non-switch) occur in the verification tasks, given an opportunity to explore their combined effects on performance. Based on the literature reviewed above, we can expect both a switch cost and a negation cost in terms of longer response times and reduced accuracy. Most importantly, an interaction between the two factors is possible; for instance, the cost of negation could be reduced (primed) or increased (interfered) in the context of language switch, compared to the language non-switch. If so, this would suggest that the two processes share resources from the same inhibitory control mechanism.

## Methods

### Participants

A total of 121 psychology students from the University of La Laguna voluntarily participated in the current study. All the participants were neurologically healthy and right-handed with normal or corrected-to-normal eyesight. They were given informed consent and received course credit for their participation. Spanish is their native language (L1) and they use English as the second language (L2). Three participants were excluded for choosing “I find it difficult to understand most of the sentences.” In a post-survey. The final sample consisted of 118 participants (98 females, *M* = 20.4 years, SD = 5.12).

To assess the participants’ language proficiency, we inquired about the age of L2 acquisition (AoA), and administered a self-rated language skills questionnaire, in which participants rated on a five-point scale their own-perceived L1 (Spanish) and L2 (English) knowledge, with 5 indicating excellent and 1, poor. All participants reported having an L2 level higher than the B1 in the CEFR test or an equivalent level in other English tests. As illustrated in Table 1, the self-rated questionnaire confirmed that the participants were unbalanced bilinguals with significantly higher proficiency in Spanish, than in English (*t* = 19.811, *p* < 0.001). The average age of L2 acquisition (AoA) was 5.05-year-old.

**TABLE 1:** Characteristics of participants.

	Group 1: Spanish context		Group 2: English context	
SELF-RATING	L1 (Spanish)	L2 (English)	L1 (Spanish)	L2 (English)
AOA		5.60 (2.88)		4.62 (1.74)
LISTENING	4.79 (0.45)	3.66 (0.95)	4.83 (0.48)	3.42 (1.02)
SPEAKING	4.58 (0.63)	3.26 (0.83)	4.71 (0.63)	3.18 (0.91)
READING	4.74 (0.48)	3.96 (0.80)	4.80 (0.44)	3.69 (0.78)
WRITING	4.47 (0.69)	3.13 (0.78)	4.60 (0.55)	3.17 (0.83)
MEAN	4.64 (0.58)	3.50 (0.90)	4.73 (0.54)	3.37 (0.92)

### Materials

The experimental task was composed of a study phase and a verification phase. The study phase required participants to read four stories involving two protagonists (Montse and Jordi), describing their main personal traits and their activities in four different scenarios: daily life in the university, vacations, going to the beach, and a birthday party. Each story included 44–46 items each (*M* = 45.25), among which, 36 were about the protagonists. These experimental items consisted of 18 semantically related pairs, with each member of a pair assigned to one of the protagonists (e.g., Montse studies psychology, Jordi studies computer sciences). The remaining items were fillers (*M* = 9.25) to make the story natural and coherent (e.g., Then Montse and Jordi met in the library to study for a while). There were two versions of the stories written in Spanish (L1) and English (L2), respectively, although with identical content.

The verification phase was composed of 104 “wh” questions in total. Each story was followed by 26 questions, 18 of which referred to the experimental items shown in the preceding story (e.g., “Who studies psychology?”), and were followed by the name of one of the characters in a separate frame (e.g., Montse). The participants had to judge whether the name was a correct answer to the question, pressing the “yes” or the “no” response button. The remaining 8 questions referred to the filler items (e.g., Where did Montse and Jordi meet to study?). Of the experimental questions, 12 were non-switching questions asked in the same language as the initial story, and 6 were switching questions asked in the other language. The filler questions were always formulated in the same language as the story. All the questions were presented in pseudo-random order. For each story block, the first two questions were always fillers. The switching questions were always followed by 2–4 non-switching questions. Within each context language group, we created 8 counterbalanced lists resulting from 1) the facts attributed to the protagonists in the stories; 2) the facts asked in the verification questions 3) the response polarity.

### Design and procedure

The experimental design was composed of Language Sequence (switch vs non-switch), and Response Polarity (affirmative vs negative), as within-subject factors, and Context Language (L1 vs L2) as a between-subject factor. Non-switch questions were in the same language as the context story and were preceded by a question in that language (L1 → *L1*, in L1 context, or L2 → *L2* in an L2 context), while the switch questions were in a different language from the context and were preceded by at least 2 questions in the context language (L1, L1 → *L2*, in the context of L1, or L2, L2 → *L1*, in the context of L2).

Due to the COVID-19 situation, the experiment was programmed and conducted online, using the Psytoolkit toolkit (Stoet, 2010, 2017). The participants were randomly and automatically assigned to the L1 or L2 story context. Fifty-three participants received most of the linguistic materials in Spanish (L1 Context), while the remaining 65 were assigned to English materials (L2 Context). A posterior test showed that the two context groups had similar language proficiency measures (see Table 1), according to the independent samples Mann–Whitney *U* tests: age of L2 acquisition [U(116) = 2,038.000, *p* = 0.083], L2 proficiency [Reading: U(116) = 2,058.500, *p* = 0.053, Writing: U(116) = 1,697.500, *p* = 0.887, Speaking: U(116) = 1,799.500, *p* = 0.660; Listening: U(116) = 1,941.500, *p* = 0.214; Average: U(116) = 1,929, *p* = 0.262] and L1 proficiency [Reading: U(116) = 1,618.500, *p* = 0.430; Writing: U(116) = 1,585.000, *p* = 0.389; Speaking: U(116) = 1,518.500, *p* = 0.157; Listening: U(116) = 1,620.500, *p* = 0.379; Average: U(16) = 1,527, *p* = 0.253].

Participants received an email with the experiment link and were instructed to complete the experiment online on a computer and a keyboard in a quiet room, previously turning off the mobile phone to avoid distractions. In the study phase, participants were first instructed to read the story carefully, keeping in mind that there would be related questions later. Then, the story was freely read by the participants in 4 self-paced paragraphs with 8–15 sentences in each paragraph (see Supplementary material 3). In the verification phase, each trial started with a 500 ms fixation in the center of the screen, followed by a question, which remained on the screen for 3,000 ms. Next, the protagonist’s name was presented on the screen. Participants were prompted to press the “yes” response (the “j” key) or the “no” response (the “f” key) as fast and accurately as possible according to the initial story. If they failed to respond in the 5,000 ms, the program moved to the next sentence. The next trial started after a random blank period (1,000–1,200 ms). Participants were questioned on how well they had understood the story when they finished the experimental task. The questions were in a three-point scale: 1. I understand practically everything; 2. Moderate, I got lost with a few sentences; 3. Low, I find it difficult to understand most of the sentences.

### Statistical analysis

To avoid language alternation influence on the non-switching level of the Language Sequence condition, the first one non-switching question following a switching question was excluded from the statistical analysis. Nine participants and two items were excluded from the data analysis due to their high number of errors (> 40%). In addition, for each participant, verification trials with an incorrect response were excluded from the reaction times (RTs) analyses, as well as responses below 200 ms or above 2.5 standard deviations of the mean. Linear mixed-effect models (LMEMs) from the UllRtoolbox package were used to analyze the resulting trimmed RTs (R Core Team, 2015; Hernández, 2017), after normalizing with an inverse transformation (Box and Cox, 1964). Subjects and items were treated as random intercepts. Context Language, Language Sequence, and Response Polarity, as well as their interactions, were treated as fixed effects (Baayen et al., 2008). Before running the model, R-default treatment contrasts were automatically set to sum-to-zero contrasts. The structure of the estimated model employed to analyze the fixed-effects was: mod1.p = RT.p ∼ context language*response polarity*question language + (question language | sujeto) + (1 | item). More complex models including all relevant random structures were used in our initial analyses, but the models with more complex random structures failed to reliably converge (Barr, 2013). We called the Car package (Fox and Weisberg, 2018) with the function *car: Anova* (χ*2 variant)* to test significance and compute *p* values for the fixed-effects, avoiding issues of estimating denominator degrees of freedom in unbalanced designs, both mathematical and computational [see Alday et al. (2017), for an overview on parameter estimations and model fitting of LMEMs]. Since non-normality affects only the estimate of standard errors (and hence the significance of the contrasts), but not the fixed effects, a model using raw RTs was employed to extract mean differences to conduct *post hoc* contrasts.

For accuracy data, logistic regression models were estimated using as well the UllRtoolbox package (R Core Team, 2015; Hernández, 2017). Again, subjects and items were treated as random intercepts, while Context language, Language Sequence and Response Polarity, as well as their interactions, were treated as fixed effects (Baayen et al., 2008). The model used to analyze the fixed effects had the following structure: mod.hit = accuracy ∼ context language*response polarity*question language + (response polarity| sujeto) + (1| item). Type III Wald chi-square tests were adopted to test for significance and to calculate *p* values.

## Results

**FIGURE 1:**
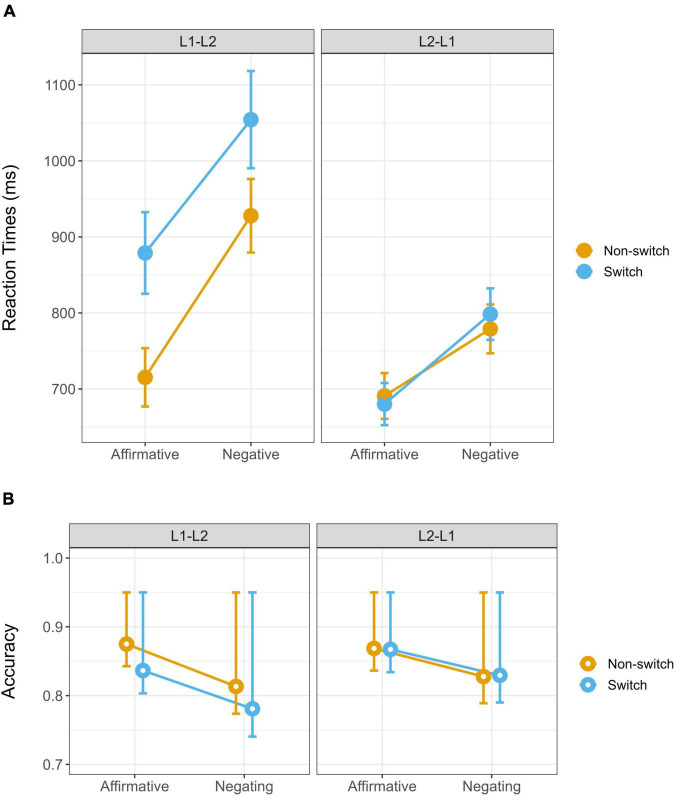
Mean RTs **(A)** and accuracy **(B)** for response polarity (affirmative vs. negative) language sequence (switch vs. non-switch) and context language (L1 vs. L2).

The average RTs for correct trimmed response time and the percentage accuracy rates across conditions are shown in Figure 1 and Table 2. The RTs analysis revealed significant effects of Response Polarity [χ2(1) = 381.50, *p* < 0.0001], Language Sequence [χ2(1) = 8.30, *p* = 0.004], and Context Language [χ2(1) = 5.43, *p* = 0.020]. These effects reflected longer RTs for: 1) negation than affirmation responses, 2) switch than non-switch questions, and L1 than L2 contexts. The two-way interactions between Response Polarity and Language Sequence did not reach significance [χ2(1) = 1.55, *p* = 0.213]. However, Context Language interacted significantly with Response Polarity [χ2(1) = 6.76, *p* = 0.009] and with Language Sequence [χ2(1) = 23.75, *p* < 0.0001]. *Post hoc* analyses revealed larger costs for language switch (the difference between switch and non-switch trials) when L1 was the main language (L1 context) (β = −152.66, SE = 28.4, *z* = −5.370, *p* < 0.0001) than when it was L2 (β = −1.79, SE = 26.7, *z* = −0.067, *p* = 0.947). Similarly, negation cost was larger in L1 context (β = −193, SE = 18.6, *z* = −10.393, *p* < 0.0001) than L2 context (β = −108, SE = 16.9, z = −6.429, *p* < 0.0001). More importantly, the three-way interaction between Response Polarity, Language Sequence and Context Language reached also significance (χ2(1) = 5.29, p = 0.021) (See Figure 1A). Given the significant three-way interaction, our initial interest was to examine how Language Sequence and Response Polarity are processed in the Context Language L1 and L2. *Post hoc* analyses showed that, for the non-switch sequence, responding “yes” took similar time in the L1 Context and in the L2 Context; however, responding “no” took longer in L1 Context than in L2 Context (Affirmative: β = −0.309, SE = 1.39, z = −0.222, *p* = 0.824; Negative: β = −3.146, SE = 1.40, z = −3.642, *p* = 0.0003). Regarding the switch sequence, the two Context Languages differed significantly both when producing a “yes” and a “no” response (Affirmative: β = −5.133, SE = 1.44, *z* = −3.571, *p* = 0.0004; Negative: β = −5.269, SE = 1.45, *z* = −3.642, *p* = 0.0003), with a longer response time in L1 Context compared to L2 Context.

**TABLE 2:** Mean RTs (ms) and ACCs (%) in the Affirmative and Negative responses deposed by Language Sequence and Context Language.

		L1 Context Language		L2 Context Language	
		Switch	Non-switch	Switch	Non-switch
RT	Affirmative	883 (597)	716 (439)	677 (340)	694 (375)
	Negative	1,057 (681)	928 (539)	801 (405)	790 (391)
ACC	Affirmative	83.7 (37.0)	87.5 (33.1)	86.7 (34.0)	86.9 (33.8)
	Negative	78.1 (41.4)	81.3 (39.0)	82.9 (37.6)	82.8 (37.8)

To better understand the three-way interaction, further analyses with the same LMEMs strategy as above was performed for each Context Language group separately. Fixed effects were reported for each Context Language group. For the L2 context group, there was a main effect of Response Polarity (β = −4.741, SE = 0.526, *t* = −9.010, *p* < 0.0001), indicating longer RTs for “no” than “yes” responses. However, for this group, neither Language Sequence (β = 0.149, SE = 0.731, *t* = 0.205, *p* = 0.838) nor the interaction reached significance (β = −0.611, SE = 0.750, *t* = −0.815, *p* = 0.415). In contrast, for the L1 context group, there were main effects of Response Polarity (β = −7.638, SE = 0.647, *t* = −11.806, *p* < 0.0001), and Language Sequence (β = −4.802, SE = 0.855, *t* = −5.620, *p* < 0.0001), which reflected longer RTs for negation than affirmation, and for switch than non-switch questions. Most importantly, the interaction between these two factors was significant for the L1 context group (β = 2.328, SE = 0.932, *t* = 2.499, *p* = 0.013), indicating relatively diminished switch cost for “no” responses in comparison to “yes” responses. All these findings indicated that the RT did not differ at the “baseline” condition (responding “yes” to non-switch questions), but the response time was highly and differentially modulated by Response Polarity and Language Sequence in both L1 and L2 groups.

For the accuracy rate logistic regression model, only the main effect of polarity resulted in significant, [χ2(1) = 12.79, *p* < 0.01], with higher accuracy for “yes” than “no” responses (see Figure 1B). The absence of the main effects of Language Sequence and Context Language as well as the interactions might be attributed to certain ceiling effect. The accuracy performance of encoding and retrieving the initial stories were similar for both groups of participants due to the ease of understanding the materials. Tables resuming the main results are viewable in Supplementary Tables 1, 2.

## Discussion

The present study investigated for the first time whether bilinguals’ language switch modulates negation processing. To this end, we tested bilingual Spanish-English speakers using a two-step encoding and verification memory-based task. The encoding phase involved reading stories that were shown in the participants’ native language (Spanish), or in their second language (English). The language switch was induced during the verification phase, by presenting the questions in the same language (non-switch) or in the alternative language (switch) as the main story. On the other hand, responses to both types of verification questions were affirmative (yes) or negative (no), so the polarity was not a feature of the sentences themselves, but rather arose during the response production. An unexpected result was that the verification times for the L1 Context were longer than for the L2 Context, which seems to be at odds with the well-known fact that unbalanced bilinguals are usually more efficient at processing their native language rather than a second language. However, this result is misleading if we neglect the interactive effects of Language Context with Response polarity and Language Sequence. Thus, if we focus on the baseline condition (non-switch and affirmative) the response time and accuracy did not differ for both language contexts. However, beyond the baseline, the RTs were modulated differently in the L1 Context (both by switching and negative polarity) and in the L2 Context (only by negative polarity). The main results of the modulation were as follows. First, an asymmetric language switch effect was found. That is, in the context of L1 (Spanish), switching to L2 in the verification involved more cognitive cost (slower and less accurate responses) than keeping the same language (L1-L1). However, in the context of L2 (English) switching to L1 did not imply an additional cognitive cost compared to keeping the same language (L2-L2). Secondly, a classical negation effect was observed, with a longer reaction time to produce negative (“no”) than affirmative responses (“yes”). Finally, although the negation effect was patent for the L1 and L2 context groups, negation only interacted with language switching in the former. That is, in the context of L1, the cost of switching from L1 to L2 was reduced for “no” responses in comparison to “yes” responses, implying a sort of priming, which is, producing negative responses benefits from a language switching sequence. These results will be discussed in detail below.

### Asymmetry of language switch cost

The finding of switching costs from L1 to L2 is consistent with the research on language-dependent differences in memory retrieval processes (Marian and Neisser, 2000; Marian and Fausey, 2006; Marian and Kaushanskaya, 2007; Wang, 2022). Such research revealed that when information was encoded in L1, and retrieval was in L2 (switch), the recall was impaired compared to using L1 in retrieval (non-switch). However, the switching cost was attenuated (found in accuracy but not speed) when encoding was in L2 and retrieval in L1 (switch) compared to using L2 both at encoding and retrieval (non-switch) (Larsen et al., 2002; Matsumoto and Stanny, 2006; Marian and Kaushanskaya, 2007). Another important finding in this literature is that proficiency moderates the effect on retrieval speed. Unbalanced bilinguals who are more proficient in one of the languages show a stronger switching effect when encoding is in the dominant language, with no language switch effect when encoding in the second language. Overall, this latency pattern coincided with that obtained in our study, with a sample of unbalanced bilinguals.

The switch cost asymmetry might be attributed to the usual direction of translation. As suggested by Marian and Kaushanskaya (2007), unbalanced bilinguals are more likely to mentally translate the less proficient language into the more proficient language (Dornic, 1978; Schrauf, 2002), resulting in stronger connections from L2 to L1 than from L1 to L2 (Kroll et al., 2002). In our case, switching from L2 to L1 was consistent with the most proficient direction of translation, resulting in the absence of language-dependent effects. A complementary explanation is that unbalanced Spanish-English participants, like ours, have more linguistic experience in retrieving information in Spanish from an English source than retrieving in English from a Spanish source, presenting an advantage for the verification in the L2-L1 direction in comparison to the L1-L2 direction (Marian and Fausey, 2006). In sum, our results confirmed a strong switching cost in the L1-L2 direction, and the absence of switching cost in the direction L2-L1 in a memory-based language-switching task.

The asymmetry of switching cost obtained here (poorer performance when switching from L1 to L2 than from L2 to L1) contrasts with the commonly reported pattern in language switching studies using naming tasks (Meuter and Allport, 1999). In these cases, the language switch asymmetry for unbalanced bilinguals was the opposite; that is, higher language-switching cost from L2 to L1 than the other way around. This is because naming in the non-dominant language requires more active inhibition on the dominant language and exerts negative priming on the following L1 trial. In contrast, little suppression is needed in the reversed direction (Green, 1998; Meuter and Allport, 1999; Finkbeiner et al., 2006; Declerck and Philipp, 2015a). Notably, the highest L1-L2 switching cost was mainly found in the lexical tasks of picture naming, while the highest L2-L1 switching cost, as the reported in the present study, is usually based on two-step memory tasks, involving more complex linguistic materials during the encoding phase (stories) and the delayed memory phase (sentence verification). In fact, numerous empirical studies have demonstrated that task-dependent factors might influence language transition cost, such as sentential context (Declerck and Philipp, 2015b), contextually changing language proficiency (Bonfieni et al., 2019), grammatical structure (Gollan and Goldrick, 2016), or cue-to-stimulus intervals (Verhoef et al., 2009), etc.

### Language switch and negation

Consistent with the previous work (Clark and Chase, 1972; Carpenter and Just, 1975; Zhang et al., see text footnote 1), we found lower accuracy and longer RTs for negative than affirmative responses in both L1 and L2 context groups, suggesting that more elaboration and more cognitive resources were required for producing negative responses compared to producing affirmative responses. However, the major finding of the present study was the interaction between language sequence (switch vs. non-switch) and response polarity (affirmative vs. negative) in the most demanding switching context (from L1 to L2). Specifically, results showed that the switching cost from L1 to L2 diminished for negative responses compared to affirmative responses, indicating a priming of directional switch over negation. A statistical interaction between two variables in a reaction time task, in this case language switching and linguistic negation, may indicate that the processes underlying these variables share neurocognitive resources (Sternberg, 1998). In fact, there is independent evidence that language switch in bilinguals and processing negation utilize the general-domain mechanisms of inhibitory control.

Language switch has been described as a conflict monitoring process, since the bilinguals must be able to actively inhibit one language while using the other, to minimize interference. Neuroimaging studies have provided evidence that inhibitory control networks, including the anterior cingulate, the SMA or the prefrontal gyrus are recruited during language switch (Guo et al., 2011; Abutalebi et al., 2012; de Bruin et al., 2014). Moreover, in recent years, there is an emerging view that negation causes conceptual suppression by recruiting inhibitory mechanisms, particularly those concerned with preventing or stopping dominant reactions and representations (de Vega et al., 2016; Beltrán et al., 2019; Liu B. et al., 2020; Dudschig et al., 2021; Montalti et al., 2021). More relevant to our study, the denial function of negation is also empirically associated with inhibition effects (Cornish and Wason, 1970; Craik and Tulving, 1975; Fiedler et al., 1996; Mayo et al., 2014; Zhang et al., see text footnote 1). Specifically, the production of correct negative responses in the verification phase of a memory task impairs the long-term memory of the negated contents compared to the production of correct affirmative responses. The underlying mechanism of this amnesia effect of denial could also be attributed to the recruitment of inhibition, similar to the case of sentential negation (Mayo et al., 2014; Zhang et al., see text footnote 1). Based on these two accounts, we can interpret the reduced cost of negation in the context of language switching as supporting the idea that the two processes recruit the same neurocognitive mechanism of inhibitory control, producing a kind of priming effect. In other words, a question switching to the target (especially from L1 to L2) induces a strong inhibition state that could facilitate the inhibition-demanding negative responses. Hence, there is no need to reactivate the mechanism, and the negating response was facilitated. Note that we examined here the polarity effects in the production of affirmative or negative responses, rather than the comprehension of sentences differing in polarity, as frequently is done in other studies on the inhibitory effect of the negation (de Vega et al., 2016; Beltrán et al., 2018, 2019; Liu B. et al., 2020). It is possible that the language-switching priming effect on negation is confined to the production of negative responses, whereas no such effect would be obtained for the comprehension of negative sentences. The issue of how language switching and sentential negation influence each other requires further investigation. Although this measure did not provide unequivocal evidence that inhibition is the only mechanism under language control and negation, it shows the possibility that inhibition may explain at least part of the shared mechanism of language control processing and negation processing.

### Conclusion and further avenues

This is the first study, to our knowledge, that examined how two apparently unrelated linguistic processes (language switching and producing negative answers) modulate each other in different language contexts. The choice of the two processes was motivated by the hypothesis that they recruit inhibitory control resources, and therefore they could interact when combined in the same task. The results found asymmetric switch cost (L1 to L2 > L2 to L1), negation cost (negation > affirmation), and interactive effects between them, which are suggestive of shared processes. This could have implications for theoretical and applied research fields, for instance, implement methods to learn a second language, better understand decision making processes, study inhibitory control disorders, long-term memory processes, etc. However, the current study has some limitations. First, despite the fact that the two groups of participants submitted to the L1 and L2 contexts, respectively, did not differ significantly in language proficiency and despite adopting the same material contents in Spanish and English for the study phase and verification phase, the between-group design of language context could induce biased results. Future studies are needed to adopt a within-participant design to better control for these possible biases. Second, future studies are needed to clarify whether the observed interactions between response polarity and language switch involves specific inhibitory control networks in the brain (e.g., SMA, rIFG, anterior cingulate cortex), using neuroimaging, EEG and non-invasive brain stimulation. Also, it might be useful to test these interactive effects with different task demands and materials, including sentential negation, naming paradigms, etc.

## Data availability statement

The original contributions presented in this study are included in the article/Supplementary material, further inquiries can be directed to the corresponding author.

## Ethics statement

The studies involving human participants were reviewed and approved by Comité de Ética de la Investigación y Bienestar Animal Vicerrectorado de Investigación y Transferencia de Conocimiento Universidad de La Laguna, La Laguna. Email: ceiba@ull.es. The patients/participants provided their written informed consent to participate in this study.

## Author contributions

AZ, MV, and DB contributed to the conception and design of the study. AZ and YF performed the statistical analysis. AZ wrote the first draft of the manuscript. MV and DB wrote sections of the manuscript. AZ, MV, YF, HW, and DB contributed to the manuscript revision, read, and approved the submitted version. All authors contributed to the article and approved the submitted version.

## Footnotes

1 Zang, A., Beltrán, D., Wang, H., González, K. R., and de Vega, M. (submitted). *Does negation-induced forgetting result from inhibition or associative interference?* Available online at: http://dx.doi.org/10.2139/ssrn.4108574.

## References

[B1] Abutalebi J. Della Rosa P. A. Green D. W. Hernandez M. Scifo P. Keim R. (2012). Bilingualism tunes the anterior cingulate cortex for conflict monitoring. *Cereb. Cortex* 22 2076–2086. 10.1093/cercor/bhr287 22038906

[B2] Abutalebi J. Green D. W. (2008). Control mechanisms in bilingual language production: Neural evidence from language switching studies. *Lang. Cogn. Process*. 23, 557–582.

[B3] Alday P. M. Schlesewsky M. Bornkessel-Schlesewsky I. (2017). Electrophysiology reveals the neural dynamics of naturalistic auditory language processing: event- related potentials reflect continuous model updates. *ENeuro* 4:ENEURO.0311–16.2017, 10.1523/ENEURO.0311-16.2017 29379867 PMC5779117

[B4] Aravena P. Delevoye-Turrell Y. Deprez V. Cheylus A. Paulignan Y. Frak V. (2012). Grip force reveals the context sensitivity of language-induced motor activity during “Action Words” processing: evidence from sentential negation. *PLoS One* 7:e50287. 10.1371/journal.pone.0050287 23227164 PMC3515598

[B5] Baayen R. H. Davidson D. J. Bates D. M. (2008). Mixed-effects modeling with crossed random effects for subjects and items. *J. Mem. Lang.* 59 390–412. 10.1016/j.jml.2007.12.005

[B6] Barr D. J. (2013). Random effects structure for testing interactions in linear mixed-effects models. *Front. Psychol.* 4:328. 10.3389/fpsyg.2013.00328 23761778 PMC3672519

[B7] Bartoli E. Tettamanti A. Farronato P. Caporizzo A. Moro A. Gatti R. (2013). The disembodiment effect of negation: negating action-related sentences attenuates their interference on congruent upper limb movements. *J. Neurophys.* 109 1782–1792. 10.1152/jn.00894.2012 23307950

[B8] Beltrán D. Liu B. de Vega M. (2021). Inhibitory mechanisms in the processing of negations: a neural reuse hypothesis. *J. Psychol. Res.* 50 1243–1260. 10.1007/s10936-021-09796-x 34383177 PMC8660707

[B9] Beltrán D. Morera Y. García-Marco E. De Vega M. (2019). Brain inhibitory mechanisms are involved in the processing of sentential negation, regardless of its content. Evidence from EEG theta and beta rhythms. *Front. Psychol.* 10:1782. 10.3389/fpsyg.2019.01782 31440181 PMC6694754

[B10] Beltrán D. Muñetón-Ayala M. de Vega M. (2018). Sentential negation modulates inhibition in a stop-signal task. Evidence from behavioral and ERP data. *Neuropsychologia* 112 10–18. 10.1016/j.neuropsychologia.2018.03.004 29518413

[B11] Bialystok E. Klein R. Craik F. I. M. Viswanathan M. (2004). Bilingualism, aging, and cognitive control: evidence from the Simon task. *Psychol. Aging* 19 290–303. 10.1037/0882-7974.19.2.290 15222822

[B12] Bloom L. M. (1970). *Language Development: Form and Function in Emerging Grammars.* Cambridge, MA: MIT Press.

[B13] Bonfieni M. Branigan H. P. Pickering M. J. Sorace A. (2019). Language experience modulates bilingual language control: the effect of proficiency, age of acquisition, and exposure on language switching. *Acta Psychol.* 193 160–170. 10.1016/j.actpsy.2018.11.004 30640064

[B14] Box G. E. P. Cox D. R. (1964). An Analysis of Transformations. *J. R. Statist. Soc.* 26 211–243. 10.1111/j.2517-6161.1964.tb00553.x

[B15] Bultena S. Dijkstra T. Van Hell J. G. (2015). Language switch costs in sentence comprehension depend on language dominance: evidence from self-paced reading. *Bilingualism* 18 453–469. 10.1017/S1366728914000145

[B16] Carpenter P. A. Just M. A. (1975). Sentence comprehension: a psycholinguistic processing model of verification. *Psychol. Rev.* 82 45–73. 10.1037/h0076248

[B17] Choi S. (1988). The semantic development of negation: a cross-linguistic longitudinal study. *J. Child Lang.* 15 517–531. 10.1017/S030500090001254X 3198720

[B18] Clark H. H. Chase W. G. (1972). On the process of comparing sentences against pictures. *Cogn. Psychol.* 3 472–517. 10.1016/0010-0285(72)90019-9

[B19] Cornish E. R. Wason P. C. (1970). The Recall of Affirmative and Negative Sentences in an Incidental Learning Task. *Quart. J. Exp. Psychol.* 22 109–114. 10.1080/00335557043000032

[B20] Costa A. Hernández M. Sebastián-Gallés N. (2008). Bilingualism aids conflict resolution: evidence from the ANT task. *Cognition* 106 59–86. 10.1016/j.cognition.2006.12.013 17275801

[B21] Costa A. Santesteban M. (2004). Lexical access in bilingual speech production: evidence from language switching in highly proficient bilinguals and L2 learners. *J. Mem. Lang.* 50 491–511. 10.1016/j.jml.2004.02.002

[B22] Craik F. I. Tulving E. (1975). Depth of processing and the retention of words in episodic memory. *J. Exp. Psychol.* 104 268–294.

[B23] Davies G. M. Thomson D. M. (1988). *Memory in Context: Context in Memory.* Hoboken: John Wiley & Sons.

[B24] De Bot K. (1992). A bilingual production model: levelt’s ‘speaking’model adapted downloaded from. *Appl. Ling.* 13 1–24.

[B25] de Bruin A. Roelofs A. Dijkstra T. FitzPatrick I. (2014). Domain-general inhibition areas of the brain are involved in language switching: FMRI evidence from trilingual speakers. *NeuroImage* 90 348–359. 10.1016/j.neuroimage.2013.12.049 24384153

[B26] de Vega M. Morera Y. León I. Beltrán D. Casado P. Martín-Loeches M. (2016). Sentential negation might share neurophysiological mechanisms with action inhibition. Evidence from frontal theta rhythm. *J. Neurosci.* 36 6002–6010. 10.1523/JNEUROSCI.3736-15.2016 27251621 PMC6601810

[B27] Declerck M. Philipp A. M. (2015b). A sentence to remember: instructed language switching in sentence production. *Cognition* 137 166–173. 10.1016/j.cognition.2015.01.006 25659539

[B28] Declerck M. Philipp A. M. (2015a). A review of control processes and their locus in language switching. *Psychon. Bull. Rev.* 22 1630–1645. 10.3758/s13423-015-0836-1 25917142

[B29] Declerck M. Philipp A. M. Koch I. (2013). Bilingual control: sequential memory in language switching. *J. Exp. Psychol.* 39 1793–1806. 10.1037/a0033094 23773181

[B30] Dornic S. (1978). “The bilingual’s performance: Language dominance, stress, and individual differences,” in *Language Interpretation and Communication*, eds Gerver D. Sinaiko H. W. (New York: Plenum), 259–271.

[B31] Dudschig C. Kaup B. Svaldi J. Gulewitsch M. D. (2021). Negation processing in children with ADHD: the generic problem of using negation in instructions. *J. Psychol. Res.* 50 1309–1320. 10.1007/s10936-021-09789-w 34374888 PMC8660710

[B32] Fiedler K. Walther E. Armbruster T. Fay D. Naumann U. (1996). Do you really know what you have seen? Intrusion errors and presuppositions effects on constructive memory. *J. Exp. Soc. Psychol.* 32 484–511. 10.1006/jesp.1996.0022

[B33] Finkbeiner M. Almeida J. Janssen N. Caramazza A. (2006). Lexical selection in bilingual speech production does not involve language suppression. *J. Exp. Psychol.* 32 1075–1089. 10.1037/0278-7393.32.5.1075 16938047

[B34] Fox J. Weisberg S. (2018). Visualizing fit and lack of fit in complex regression models: effect plots with partial residuals. *J. Statist. Softw.* 87 1–27. 10.18637/jss.v087.i09

[B35] Gade M. Declerck M. Philipp A. M. Rey-Mermet A. Koch I. (2021). Assessing the evidence for asymmetrical switch costs and reversed language dominance effects – a meta-analysis. *J. Cogn.* 4:55. 10.5334/JOC.186 34611575 PMC8447966

[B36] García-Marco E. Morera Y. Beltrán D. de Vega M. Herrera E. Sedeño L. (2019). Negation markers inhibit motor routines during typing of manual action verbs. *Cognition* 182 286–293. 10.1016/j.cognition.2018.10.020 30390568

[B37] Gollan T. H. Goldrick M. (2016). Grammatical constraints on language switching: language control is not just executive control. *J. Mem. Lang.* 90 177–199. 10.1016/j.jml.2016.04.002 27667899 PMC5033271

[B38] Green D. W. (1998). Mental control of the bilingual lexico-semantic system. *Bilingualism* 1 67–81. 10.1017/s1366728998000133

[B39] Guo T. Liu H. Misra M. Kroll J. F. (2011). Local and global inhibition in bilingual word production: FMRI evidence from Chinese–English bilinguals. *NeuroImage* 56 2300–2309. 10.1016/j.neuroimage.2011.03.049 21440072 PMC3741343

[B40] Hasegawa M. Carpenter P. A. Just M. A. (2002). An fMRI study of bilingual sentence comprehension and workload. *NeuroImage* 15 647–660. 10.1006/nimg.2001.1001 11848708

[B41] Hernández J. (2017). *ULLRToolbox for R (Version 1.0). [Software]*. Available online at: https://sites.google.com/site/ullrtoolbox/00-instalacion-windows (accessed November 10, 2020).

[B42] Kaup B. Zwaan R. A. (2003). Effects of Negation and Situational Presence on the Accessibility of Text Information. *J. Exp. Psychol.* 29 439–446. 10.1037/0278-7393.29.3.439 12776754

[B43] Kroll J. F. Michael E. Tokowicz N. Dufour R. (2002). The development of lexical fluency in a second language. *Sec. Lang. Res.* 18 137–171. 10.1191/0267658302sr201oa

[B44] Kroll J. F. Dussias P. E. Bice K. Perrotti L. (2015). Bilingualism, mind, and brain. *Annu. Rev. Linguist*. 1:377. 28642932 10.1146/annurev-linguist-030514-124937 PMC5478196

[B45] Larsen S. F. Schrauf R. W. Fromholt P. Rubin D. C. (2002). Inner speech and bilingual autobiographical memory: a Polish-Danish cross-cultural study. *Memory* 10 45–54. 10.1080/09658210143000218 11747575

[B46] Liu B. Gu B. Beltrán D. Wang H. de Vega M. (2020). Presetting an inhibitory state modifies the neural processing of negated action sentences. An ERP study. *Brain Cogn.* 143:105598. 10.1016/j.bandc.2020.105598 32645511

[B47] Liu H. Tong J. de Bruin A. Li W. He Y. Li B. (2020). Is inhibition involved in voluntary language switching? Evidence from transcranial direct current stimulation over the right dorsolateral prefrontal cortex. *Int. J. Psychophysiol.* 147 184–192. 10.1016/j.ijpsycho.2019.12.002 31830498

[B48] MacDonald M. C. Just M. A. (1989). Changes in Activation Levels With Negation. *J. Exp. Psychol.* 15 633–642. 10.1037/0278-7393.15.4.633 2526856

[B49] Marian V. Fausey C. M. (2006). Language-dependent memory in bilingual learning. *Appl. Cogn. Psychol.* 20 1025–1047. 10.1002/acp.1242

[B50] Marian V. Kaushanskaya M. (2007). Language context guides memory content. *Psychon. Bull. Rev.* 14 925–933. 10.3758/BF03194123 18087961

[B51] Marian V. Neisser U. (2000). Language-dependent recall of autobiographical memories. *J. Exp. Psychol.* 129 361–368. 10.1037/0096-3445.129.3.361 11006905

[B52] Matsumoto A. Stanny C. J. (2006). Language-dependent access to autobiographical memory in Japanese-English bilinguals and US monolinguals. *Memory* 14 378–390. 10.1080/09658210500365763 16574592

[B53] Mayo R. Schul Y. Burnstein E. (2004). “I am not guilty” vs “I am innocent”: successful negation may depend on the schema used for its encoding. *J. Exp. Soc. Psychol.* 40 433–449. 10.1016/j.jesp.2003.07.008

[B54] Mayo R. Schul Y. Rosenthal M. (2014). If you negate, you may forget: negated repetitions impair memory compared with affirmative repetitions. *J. Exp. Psychol.* 143 1541–1552. 10.1037/a0036122 24635186

[B55] Meuter R. F. I. Allport A. (1999). Bilingual language switching in naming: asymmetrical costs of language selection. *J. Mem. Lang.* 40 25–40. 10.1006/JMLA.1998.2602

[B56] Montalti M. Calbi M. Cuccio V. Umiltà M. A. Gallese V. (2021). Is motor inhibition involved in the processing of sentential negation? An assessment via the Stop-Signal Task. *Psychol. Res.* 0123456789. [Epub ahead of print] 10.1007/s00426-021-01512-7 33905001 PMC9873753

[B57] Orenes I. Beltrán D. Santamaría C. (2014). How negation is understood: evidence from the visual world paradigm. *J. Mem. Lang.* 74 36–45. 10.1016/j.jml.2014.04.001

[B58] Papeo L. Hochmann J. R. Battelli L. (2016). The default computation of negated meanings. *J. Cogn. Neurosci.* 28 1980–1986. 10.1162/jocn_a_01016 27458753

[B59] Pea R. D. (1980). “The development of negation in early child language,” in *The Social Foundations of Language and thought:Essays in Honor of Jerome S. Bruner*, ed. Olson D. R. (New York, NY: W. W. Norton), 156–186.

[B60] R Core Team (2015). *R: A language and Environment for Statistical Computing.* Vienna, Austria: R Foundation for Statistical Computing.

[B61] Schrauf R. W. (2002). Bilingual inner speech as the medium of cross-modular retrieval in autobiographical memory. *Behav. Brain Sci.* 25 698–699.

[B62] Sternberg S. (1998). “Discovering mental processing stages: The method of additive factors,” in *Methods, Models, and Conceptual Issues: An Invitation to Cognitive Science*, eds Scarborough D. Sternberg S. (Cambridge: The MIT Press), 703–863.

[B63] Stoet G. (2010). PsyToolkit: a software package for programming psychological experiments using Linux. *Behav. Res. Methods* 42 1096–1104. 10.3758/BRM.42.4.1096 21139177

[B64] Stoet G. (2017). PsyToolkit: a novel web-based method for running online questionnaires and reaction-time experiments. *Teach. Psychol.* 44 24–31. 10.1177/0098628316677643

[B65] Terao Y. Ugawa Y. (2002). Basic mechanisms of TMS. *J. Clin. Neurophysiol.* 19 322–343. 10.1097/00004691-200208000-0000 12436088

[B66] Tettamanti M. Manenti R. Della Rosa P. A. Falini A. Perani D. Cappa S. F. (2008). Negation in the brain: modulating action representations. *NeuroImage* 43 358–367. 10.1016/j.neuroimage.2008.08.004 18771737

[B67] Tomasino B. Weiss P. H. Fink G. R. (2010). To move or not to move: imperatives modulate action-related verb processing in the motor system. *Neuroscience* 169 246–258. 10.1016/j.neuroscience.2010.04.039 20420884

[B68] Tulving E. Thomson D. M. (1973). Encoding specificity and retrieval processes in episodic memory. *Psychol. Rev.* 80 352–373. 10.1037/h0020071

[B69] Verhoef K. Roelofs A. Chwilla D. J. (2009). Role of inhibition in language switching: evidence from event-related brain potentials in overt picture naming. *Cognition* 110 84–99. 10.1016/j.cognition.2008.10.013 19084830

[B70] Vitale F. Monti I. Padrón I. Avenanti A. de Vega M. (2021). The neural inhibition network is causally involved in the disembodiment effect of linguistic negation. *Cortex* 147 72–82. 10.1016/j.cortex.2021.11.015 35026556

[B71] Wang L. (2022). Influences of Language Shift on Speech Fluency in Memory Production of Unbalanced Chinese-English Bilinguals. *Theory Practice Lang. Stud.* 12 375–381. 10.17507/tpls.1202.21

[B72] Wang X. (2015). Language control in bilingual language comprehension: evidence from the maze task. *Front. Psychol.* 6:1179. 10.3389/fpsyg.2015.01179 26347675 PMC4543796

