# Supplementary material for: Language switching may facilitate the processing of negative responses
Source: Front Psychol. 2022 Sep 6;13:906154. doi: 10.3389/fpsyg.2022.906154 (PMC9486385; doi:10.3389/fpsyg.2022.906154)
Supplement: Supplementary file 1 [file Table_1.DOCX]

**Supplementary**

**Supplementary 1.** *Summary of LME models conducted for RTs, followed by combined analysis of data from the Context L1 and the Context L2 separately.*

| Model 1: General Analysis (RTs)  *Factors: Context Language, Response Polarity, Language Sequence* | | | | | | | | | | | | | | | | | | | | |
| --- | --- | --- | --- | --- | --- | --- | --- | --- | --- | --- | --- | --- | --- | --- | --- | --- | --- | --- | --- | --- |
|  | Context Language | | | | | | | | | | |  | χ^2^(1) = 5.43, *p*=.020* | | | | | | | |
|  | Response Polarity | | | | | | | | | | |  | χ^2^(1) = 381.50, *p*<.0001*** | | | | | | | |
|  | Language Sequence | | | | | | | | | | |  | χ^2^(1) = 8.30, *p=*0.004** | | | | | | | |
|  | Context Language x Response Polarity | | | | | | | | | | |  | χ^2^(2) = 6.76, *p=*.009** | | | | | | | |
|  | Context Language x Language Sequence | | | | | | | | | | |  | χ^2^(1) = 23.75, *p<.*0001***** | | | | | | | |
|  | Response Polarity x Language Sequence | | | | | | | | | | |  | χ^2^(1) = 1.55, *p*=.213 | | | | | | | |
|  | Context Language x Response Polarity x Language Sequence | | | | | | | | | | |  | χ^2^(9) = 5.29, *p*=.021* | | | | | | | |
| Follow-up comparisons | | | | | | | | | | | | | | | | | | | | |
| Context Language x Response Polarity condition | | | | | | | | | | | | | | | | | | | | |
|  | | | | L1 Context | | | | | | | | |  | L2 Context | | | | | | |
| Polarity | | | | Estimate | | *SE* | | z | | *p* | | |  | Estimate | | *SE* | | z | | *p* |
| A-N | | | | -193 | | 18.6 | | -10.393 | | <.0001 | | |  | -108 | | 16.9 | | -6.429 | | <.0001 |
|  | | |  |  | |  | |  | |  | | |  |  | |  | |  | |  |
| Context Language x Language Sequence condition | | | | | | | | | | | | | | | | | | | | |
|  | | | | | L1 Context | | | | | | |  | L2 Context | | | | | | | |
| Polarity | | | | | Estimate | | *SE* | | z | | *p* |  | Estimate | | *SE* | | z | | *p* | |
| Non-switch - Switch | | | | | -152.66 | | 28.4 | | -5.370 | | <.0001 |  | -1.79 | | 26.7 | | -0.067 | | 0.947 | |
|  | |  | | |  | |  | |  | |  |  |  | |  | |  | |  | |
| Separate analysis of RT data across groups | | | | | | | | | | | | | | | | | | | | |
| Model 2: Context 1  *Factors: Response Polarity, Language Sequence* | | | | | | | | | | | | | | | | | | | | |

|  | Estimate | *SE* | t | *p* |
| --- | --- | --- | --- | --- |
| Intercept | 11668.784 | 1.113 | 10488.230 | <.0001*** |
| Polarity Negative | -7.638 | 0.647 | -11.806 | <.0001*** |
| Language Sequence Switch | -4.802 | 0.855 | -5.620 | <.0001*** |
| Polarity Negative x Language Sequence Switch | 2.328 | 0.932 | 2.499 | .013* |

| Model 3: Context 2  *Factors: Response Polarity, Language Sequence* |
| --- |

|  | Estimate | *SE* | t | *p* |
| --- | --- | --- | --- | --- |
| Intercept | 11669.070 | 1.017 | 11474.932 | <.0001*** |
| Polarity Negative | -4.741 | 0.526 | -9.010 | <.0001*** |
| Language Sequence Switch | 0.149 | 0.731 | 0.205 | .838 |
| Polarity Negative x Language Sequence Switch | -0.611 | 0.750 | -0.815 | .415 |

**Supplementary 2.** Summary of LME models conducted for ACC.

| Model 4: General Analysis (ACC)  *Factors: Context Language, Response Polarity, Language Sequence* | | | |
| --- | --- | --- | --- |
|  | Context Language |  | χ^2^(1) = 1.956, *p*=.162 |
|  | Response Polarity |  | χ^2^(1) = 12.790, *p=*.0003*** |
|  | Language Sequence |  | χ^2^(1) = 1.072, *p=*.300 |
|  | Context Language x Response Polarity |  | χ^2^(2) = 0.444, *p=*.505 |
|  | Context Language x Language Sequence |  | χ^2^(1) = 3.043, *p=.*081 |
|  | Response Polarity x Language Sequence |  | χ^2^(1) = 0.167, *p*=.683 |
|  | Context Language x Response Polarity x Language Sequence |  | χ^2^(9) = 0.077, *p*=.782 |

**Supplementary 3 An example of stimuli used in study phase and verification phase.**

**Study phase**

| **Spanish initial story (L1 Context)** | **English initial story (L2 Context)** |
| --- | --- |
| Montse y Jordi pensaban viajar juntos durante las vacaciones de verano. | Montse and Jordi planed to travel together during summer holiday. |
| Buscaron información sobre viajes en Internet. | They searched for some travelling information on the Internet. |
| Montse quería ir a Italia, | Montse wanted to go to Italy, |
| a visitar los museos. | to visit the museums. |
| Además, Montse prefería viajar en crucero. | In addition, Montse preferred to travel by cruise. |
| En cambio, Jordi quería ir a Suiza. | Instead, Jordi wanted to go to Switzerland |
| a esquiar en los Alpes, | to ski in the Alps, |
| y prefería ir en avión. | and preferred to go by plane. |
| A Montse, le gusta planificar los itinerarios del viaje, | Montse likes to plan the travel itineraries, |
| mientras que Jordi prefiere improvisar la ruta del viaje. | while Jordi prefers to improvise the travel route. |
| Finalmente decidieron ir juntos a Italia en avión. | Finally they decided to go together to Italy by plane. |
| Montse reservó el alojamiento para el viaje. | Montse booked the accommodation for the trip. |
| Jordi reservó los billetes de avión para el viaje. | Jordi booked the flight tickets for the trip. |
| Al llegar al aeropuerto, Montse compró unos chocolates para el viaje, | When they arrived at the airport, Montse bought some chocolates for the trip, |
| mientras que Jordi compró unas revistas para el viaje. | while Jordi bought some magazines for the trip. |
| Más tarde, subieron al avión después de un café rápido. | Later, they boarded the plane after a quick coffee. |
| Montse comentó que hablaría con la gente en inglés. | Montse commented that she would talk to people in English. |
| Ella quería conocer a algunos artistas. | She wanted to meet some artists. |
| Jordi dijo que él se sentía capaz de hablar en italiano. | Jordi said that he would be able to speak in Italian. |
| Quería conocer a algunos músicos. | He wanted to meet some musicians. |
| Jordi pidió un periódico a una azafata. | Jordi asked a stewardess for a newspaper. |
| Montse pidió una manta a la azafata. | Montse asked the stewardess for a blanket. |
| Cuando el avión finalmente aterrizó en el aeropuerto de Milán, no pudieron encontrar su equipaje. | When the plane finally landed at Milan airport, they couldn't find their luggage. |
| La maleta de Montse estaba decorada con pegatinas de comics. | Montse's suitcase was decorated with comic stickers. |
| Había comprada la maleta en el Carrefour. | She had bought the suitcase at the Carrefour. |
| A Montse le preocupaba perder los zapatos caros que tenía en la maleta. | Montse was worried about losing the expensive shoes she had in her suitcase. |
| Jordi llevaba una simple etiqueta de identificación en la maleta. | Jordi's had just a luggage tag on the suitcase. |
| Él había comprado la maleta en el Corte Inglés. | He had bought the suitcase at the Corte Inglés. |
| A Jordi le preocupaba perder el cepillo eléctrico que tenía en la maleta. | Jordi was worried about losing the electric toothbrush he had in his suitcase. |
| Finalmente, un asistente del aeropuerto les ayudó a encontrar el equipaje y comenzaron sus vacaciones. | Finally, an assistant at the airport helped them find the luggage and they started their vacation. |
| A Montse le pareció que Milán era una ciudad de moda. | Montse thought that Milan was a fashionable city. |
| A Jordi, en cambio, Milán le pareció una ciudad un poco fría. | Jordi, on the other hand, found Milan a bit cold. |
| Ellos visitaron el Castello Sforzesco primero. | They visited the Castello Sforzesco first. |
| Porque Jordi quería ver la última escultura de Miguel Ángel. | Because Jordi wanted to see Michelangelo's latest sculpture. |
| Montse quería ver la pintura mural de Leonardo da Vinci. | Montse wanted to see Leonardo da Vinci's wall painting |
| Jordi dijo que le gustaba sobretodo la arquitectura gótica. | Jordi said that he specially liked the gothic architecture. |
| Montse dijo que prefería la arquitectura barroca. | Montse said she preferred baroque architecture. |
| Al salir del Castello Sforzesco, descansaron en un banco. | Getting out from the Castello Sforzesco, they took a rest on a bench. |
| Entonces Montse sugirió probar los típicos helados de Milán. | Then Montse suggested trying the typical Milan ice-creams. |
| Montse se tomó un helado de yogur, | Montse had a yogurt ice cream, |
| mientras que Jordi tomó un helado de pistacho. | while Jordi took a pistachio ice cream. |
| Jordi sugirió probar la mejor pizza de Milan para cenar. | Jordi suggested trying the best pizza in Milan for dinner. |
| Para después de la cena, a Montse quería ir a la ópera, | For after dinner, Montse wanted to go to the opera, |
| mientras que a Jordi prefería ver un partido de fútbol. | while Jordi preferred to watch a soccer game. |
| Decidieron ver un partido de fútbol esta noche. | They decided to watched a soccer game this night. |

**Verification phase**

| **Spanish context question (L1 Context)** | **Answer** | **Language** | **Language Sequence** | **Polarity** |
| --- | --- | --- | --- | --- |
| Montse y Jordi pensaban viajar juntos durante las vacaciones de … | Summer | L1 | filler | Affirmative |
| ¿Dónde buscaron información sobre viajes? | On Internet | L1 | filler | Affirmative |
| Who wanted to go to Switzerland? | Montse | L2 | Switch | Negative |
| ¿Quién quería esquiar? | Jordi | L1 |  | Affirmative |
| ¿Quién prefería viajar en avión? | Jordi | L1 | Non-switch | Affirmative |
| ¿A quién le gusta planificar los itinerarios del viaje? | Jordi | L1 | Non-switch | Negative |
| ¿Con qué transporte eligieron viajar Montse y Jordi? | Plane | L1 | filler | Affirmative |
| Who booked the flight tickets for the trip. | Jordi | L2 | Switch | Affirmative |
| ¿Quién compró chocolates para el viaje? | Jordi | L1 |  | Negative |
| Montse y Jordi subieron al avión después de un… | Coffee | L1 | filler | Affirmative |
| Who felt able to talk with people in Italian? | Montse | L2 | Switch | Negative |
| ¿Quién quería conocer músicos? | Jordi | L1 |  | Affirmative |
| ¿Quién le pidió una manta a la azafata? | Montse | L1 | Non-switch | Affirmative |
| ¿Qué no se pudo encontrar después del aterrizaje? | The luggage | L1 | filler | Affirmative |
| Who had the suitcase decorated with comics stickers？ | Montse | L2 | Switch | Affirmative |
| ¿Quién compró la maleta en el Corte Inglés? | Montse | L1 |  | Negative |
| ¿Quién se preocupó por perder los zapatos caros? | Jordi | L1 | Non-switch | Negative |
| ¿Quién ayudó a Montse y Jordi a encontrar el equipaje? | An assistant | L1 | filler | Affirmative |
| ¿Quién pensó que Milán era una ciudad de moda? | Montse | L1 | Non-switch | Affirmative |
| ¿Dónde visitaron primero? | Castello Sforzesco | L1 | filler | Affirmative |
| Who wanted to see the wall painting of Leonardo da Vinci? | Jordi | L2 | Switch | Negative |
| ¿A quien le gusta la arquitectura barroca? | Montse | L1 |  | Affirmative |
| Después de salir del banquillo, ¿dónde descansaron Montse y Jordi? | On a bench | L1 | filler | Affirmative |
| ¿Quién sugirió probar la mejor pizza de Milan para cenar? | Montse | L1 | Non-switch | Negative |
| Who had a yogurt ice cream? | Montse | L2 | Switch | Affirmative |
| ¿A quién quería ver un partido de fútbol? | Montse | L1 |  | Negative |

| **English context question (L2 Context)** | **Answer** | **Language** | **Language Sequence** | **Polarity** |
| --- | --- | --- | --- | --- |
| Montse and Jordi planed to travel together during... holiday. | Summer | L2 | filler | Affirmative |
| Where did they search for travelling information? | On Internet | L2 | filler | Affirmative |
| ¿Quién quería ir de viaje a Suiza? | Montse | L1 | Switch | Negative |
| Who wanted to ski? | Jordi | L2 |  | Affirmative |
| Who prefered to travel by plane? | Jordi | L2 | Non-switch | Affirmative |
| Who likes to plan the travel itineraries? | Jordi | L2 | Non-switch | Negative |
| By which trasportation did Montse and Jordi choose to travel? | Plane | L2 | filler | Affirmative |
| ¿Quién reservó los billetes de avión para el viaje? | Jordi | L1 | Switch | Affirmative |
| Who bought some chocolates for the trip? | Jordi | L2 |  | Negative |
| Montse and Jordi boarded the plane after a… | Coffee | L2 | filler | Affirmative |
| ¿Quién se veía capaz de hablar con la gente en italiano? | Montse | L1 | Switch | Negative |
| Who wanted to meet musicians? | Jordi | L2 |  | Affirmative |
| Who asked the stewardess for a blanket? | Montse | L2 | Non-switch | Affirmative |
| What could not be found after landing? | The luggage | L2 | filler | Affirmative |
| ¿De quien era la maleta decorada con pegatinas de comics? | Montse | L1 | Switch | Affirmative |
| Who bought the suitcase in the Corte Ingles？ | Montse | L2 |  | Negative |
| Who was worried about losing the expensive shoes? | Jordi | L2 | Non-switch | Negative |
| Who helped Montse and Jordi find the luggage? | An assistant | L2 | filler | Affirmative |
| Who thought Milan was a fashionable city? | Montse | L2 | Non-switch | Affirmative |
| Where Montse and Jordi visit firstly? | Castello Sforzesco | L2 | filler | Affirmative |
| ¿Quién quería ver la pintura mural de Leonardo da Vinci? | Jordi | L1 | Switch | Negative |
| Who likes the Baroque architecture? | Montse | L2 |  | Affirmative |
| Where did Montse and Jordi take a rest after getting out from the bench? | On a bench | L2 | filler | Affirmative |
| Who suggested trying the best pizza in Milan for dinner? | Montse | L2 | Non-switch | Negative |
| ¿Quién se tomó de un helado de yogur? | Montse | L1 | Switch | Affirmative |
| Who wanted to watch a football match? | Montse | L2 |  | Negative |
